# Novel Small Molecule Glucagon-Like Peptide-1 Receptor Agonist S6 Stimulates Insulin Secretion From Rat Islets

**DOI:** 10.3389/fphar.2021.664802

**Published:** 2021-04-29

**Authors:** Xiaohua Yang, Min Zhang, Zhihong Lu, Linping Zhi, Huan Xue, Tao Liu, Mengmeng Liu, Lijuan Cui, Zhihong Liu, Peifeng He, Yunfeng Liu, Yi Zhang

**Affiliations:** ^1^ Department of Pharmacology, Shanxi Medical University, Taiyuan, China; ^2^ Department of Pharmacy, Shanxi Medical University, Taiyuan, China; ^3^ Key Laboratory of Cellular Physiology, Ministry of Education, Shanxi Medical University, Taiyuan, China; ^4^ School of Management, Shanxi Medical University, Taiyuan, China; ^5^ Department of Endocrinology, First Hospital of Shanxi Medical University, Shanxi Medical University, Taiyuan, China

**Keywords:** virtual screening, insulin secretion, glucagon-like peptide-1 receptor (GLP-1R), intracellular calcium concentration [(Ca2+)_i_], voltage-dependent K+ (Kv) channel

## Abstract

Glucagon-like peptide-1 receptor (GLP-1R) agonist-based therapeutics for type 2 diabetes mellitus have attracted worldwide attention. However, there are challenges in the development of small molecule GLP-1R agonists owing to the complexity of ligand recognition and signal induction mechanisms. Here, we attained S6 using virtual screening and fluorescent imaging plate reader (FLIPR)-based calcium assays. The purpose of this study was to identify and characterize S6, a novel small molecule GLP-1R agonist. Data from cellular thermal shift assay (CETSA) and Bio-Layer Interferometry (BLI) indicated that S6 could bind potently with GLP-1R. Radioimmunoassay data showed that S6 potentiated insulin secretion in a glucose-dependent manner and the insulinotropic effect was mediated by GLP-1R. Calcium imaging techniques suggested that S6 elevated the intracellular calcium concentration [(Ca^2+^)_i_] by activating GLP-1R. In patch-clamp experiments, we demonstrated that S6 inhibited voltage-dependent K^+^ (Kv) channels in a GLP-1R-dependent fashion. Besides, S6 significantly prolonged action potential duration but had no effect on voltage-dependent Ca^2+^ channels. In summary, these findings indicate that S6 stimulates glucose-dependent insulin secretion mainly by acting on GLP-1R, inhibiting Kv channels, increasing (Ca^2+^)_i_. This study will provide direction for the screening and development of novel small-molecule agents targeting GLP-1R in the future.

## Introduction

The International Diabetes Federation estimates that approximately 463 million adults (20–79 years) suffer from diabetes in 2019, and this figure is predicted to increase to 578 million by 2030 and even 700 million by 2045 (CERTARA, 2014). Type 2 diabetes mellitus accounts for approximately 90% of all diabetes cases around the world (CERTARA, 2014; Saeedi et al., 2019).

Glucagon-like peptide-1 receptor (GLP-1R) agonists are promising agents for treating type 2 diabetes mellitus, since they augment glucose-dependent insulin secretion with minimized low risk of hypoglycaemia (Hansen et al., 2009; Lin and Wang, 2009; Cohen et al., 2013; Meloni et al., 2013). GLP-1R peptide agonists, such as Liraglutide and Exenatide (Madsbad, 2009; Madsbad et al., 2011), are approved for the treatment of type 2 diabetes mellitus. However, they are peptides that require administration *via* subcutaneous injection, and concerns regarding their compliance for long-term use have emerged (Elashoff et al., 2011). To our knowledge, no small molecule agents acting as GLP-1R agonists are available for clinical use (Redij et al., 2019). Hence, the development of small molecule drugs suitable for oral administration that target GLP-1R would be appropriate to circumvent this problem.

Here, our group successfully established a pharmacophore model for virtual screening based on reported GLP-1R agonists to screen the small molecule compounds in the ZINC database. Physico-chemical properties evaluation showed that hit compounds possess potential oral activity. Finally, we screened a small molecule compound, namely, S6, by cell-based calcium flux assays on fluorescent imaging plate reader (FLIPR) fluorometric imaging detection systems. Thus, it is urgent to experimentally determine. Delightingly, the early stage studies demonstrated that S6 may be a promising novel small molecule GLP-1R agonist, which will be a basis for further experimental exploration.

## Materials and Methods

### Computational Studies

Gaussian 16 software package was involved to build and optimize the compounds (Frisch et al., 2019). The pharmacophore modeling, molecular docking and virtual screening studies were performed using SYBYL-X 2.0 software (International Diabetes Federation, 2019).

### Animals and Ethics

Male Wistar rats, weighing 180 ～ 250 g, were purchased from Shanxi Provincial People’s Hospital Experimental Animal Center (Taiyuan). The rats were housed on a 12 h light/dark cycle with free access to standard rodent chow and tap water. Protocols of animal use were performed in accordance with *Guide for the Care and Use of Laboratory Animals* of Shanxi Medical University (GBT 35892-2018). In addition, the animal study was also reviewed and approved by Laboratory Animal Ethical Committee of Shanxi Medical University.

### Islets Isolation and Cells Culture

After the male Wistar rats were euthanized, islets were separated and purified through collagenase P (Roche, United States) digestion and Histopaque-1077 (Sigma-Aldrich, United States) density gradient centrifugation. Single islet β cells were isolated by dispase II (Roche, United States) digestion. Islets or β cells were cultured in RPMI 1640 medium (Hyclone, United States) containing 11.1 mM glucose, supplemented with 10% FBS (Gibco, United States), 100 µg/ml streptomycin, and 100 U/ml penicillin (Solarbio, Beijing) in a humidified incubator with 5% CO_2_ at 37°C.

Stable CHO-K1/Gα15/GLP-1R cells were obtained from Genscript (United States), and cultured in Ham’s F12 medium (Hyclone) supplemented with 10% FBS, 100 μg/ml Hygromycin B, and 200 μg/ml Zeocin in a humidified incubator with 5% CO_2_ at 37°C.

### FLIPR-Based Calcium Assays

CHO-K1/Gα15/GLP-1R cells were plated at a density of 12,500 cells per well in black, clear-bottom 384-well plates and incubated overnight at 37°C in 5% CO_2_. Probenecid solution (Sigma, United States) was prepared, the concentration of samples (Topscience Biochemical Technology Co., Shanghai) was diluted to 10 µM (Fidock, 2005) with HBSS buffer before the experiment, and the dye loading buffer was prepared according to the FLIPR Calcium 4 Assay Kit (Molecular devices, United States) operation instructions. The cell plate was taken out from the incubator, and the medium was removed completely. Dye loading buffer was added to each well, followed by incubation for 1 h at 37°C, 5% CO_2_, and equilibration to room temperature for 15 min prior to reading on FLIPR (Molecular devices). The cell plate, test samples, and GLP-1 (7-37) (positive agonist) (Genscript) were transferred into FLIPR and the changes in (Ca^2+^)_i_ were measured. All experiments were run with two parallel replicates.

### Cellular Thermal Shift Assay (CETSA)

GLP-1R expressed by Tobacco cell-free expression system were produced by GZL Bioscience Co., Ltd. (Hangzhou), and the total proteins were divided into two aliquots, with one aliquot being treated with 10 µM S6 and the other aliquot with the same volume of 1% DMSO (vol/vol). After 45 min of incubation in the chamber, total proteins were divided into smaller aliquots, then heated at different temperatures (37–65°C) using PCR (Bio-Rad, United States) for 3 min followed by cooling for 3 min at room temperature. After centrifugation at 20,000 × *g* for 20 min at 4°C, and the supernatant was analyzed by western blot analysis.

### Western Blot Analysis

Proteins were electro-blotted (20 V, 25 min) onto a Nitrocellulose membrane (Millipore, United States) after fractionation by SDS-PAGE. The membranes were blocked with 5% (w/v) non-fat dry milk for 1 h at 37°C. Primary antibodies against rabbit GLP-1R (Proteintech, Wuhan) at dilution of 1:3000 were incubated at room temperature for 2 h, followed by incubation with HRP-conjugated secondary antibodies (1:10,000, Proteintech) for 1 h. Protein bands were identified and quantified using a ChemiDicTM XRS^+^ Imaging System (Bio-Rad) with Image Lab^TM^ software (Bio-Rad). Experiments were repeated at least three times.

### Biolayer Interferometry Binding (BLI) Analysis

The different concentrations of S6 binding to GLP-1R was measured by BLI using a FortèBio Octet RED96e instrument. GLP-1R were immobilized on SSA sensors and exposed to different concentrations of S6 (3.13、6.25、12.5、25、50 µM) in PBS buffer containing 0.1% BSA (PBSF) for an association step for 60 seconds, followed by a dissociation step for 60 seconds in PBSF buffer. Data was analyzed using the SigmaPlot software 12.5. The data was fit to a 1:1 binding model to calculate an association and dissociation rate, and K_D_ (dissociation constant) was expressed by Kd/Ka ratio (Ka = rate of association, Kd = rate of disassociation).

### Insulin Secretion Assay

Each group of five islets was pre-incubated for 30 min at 37°C in 500 µl Krebs-Ringer bicarbonate-HEPES buffer (containing in mM: 128.8 NaCl, 10 HEPES, 5 NaHCO_3_, 4.8 KCl, 2.5 CaCl_2_, 1.2 MgSO_4_, 1.2 KH_2_PO_4_, 2% Albumin Bovine V (Solarbio) and 2.8 mM glucose, pH 7.4), followed by test incubation for 30 min in different glucose concentrations alone, or in the presence and absence of S6 (10 µM), Exendin (9–39) (100 nM, Aladdin, Shanghai). The content of secreted insulin in the supernatant was then measured using a radioimmunoassay kit (North Biological Technology Research Institute of Beijing).

### Patch-Clamp Experiments

Whole-cell currents in islet β cells were recorded using an EPC-10 amplifier and PULSE software from HEKA Electronik (Lambrecht, Germany). Before the experiment, β cells were cultured on coverslips in RPMI 1640 medium containing 10% FBS for 24 h. Glass pipettes with borosilicate were pulled by a two-stage vertical pipette puller (Narishige Co., Japan) and polished by MICROFORGEMF-200 (World Precision Instruments Inc., United States). The concentration of S6 used in this protocol is 10 µM. All experiments were performed at room temperature.

To record Kv currents, pipettes were filled with intracellular solution as follows (mmol/L): 140 KCl, 10 NaCl, 10 HEPES, 1 MgCl_2_, and 0.05 EGTA; pH was adjusted to 7.25 using KOH. The extracellular solution contained (mmol/L): 141.9 NaCl, 5.6 KCl, 5 HEPES, 1.2 MgCl_2_, and 11.1 glucose; pH was adjusted to 7.4 using NaOH. β cells were voltage-clamped at a holding potential of −70 mV, followed by stepwise depolarization from −70 to + 80 mV at 10 mV increments to record the outward currents.

To record inward Ca^2+^ currents, β cells were clamped at a holding potential of −70 mV; subsequently, voltage-step depolarization was used from −50 to + 30 mV in 10 mV steps. Ca^2+^ currents were recorded in an extracellular solution composed of (mmol/L): 100 NaCl, 20 tetraethylammonium chloride (TEA), 20 BaCl_2_, 5 HEPES, 1 MgCl_2_, 4 CsCl, and 3 glucose; pH was adjusted to 7.4 using NaOH. The intracellular solution contained (mmol/L): 120 CsCl, 20 TEA, 5 Mg-ATP, 1 MgCl_2_, and 0.05 EGTA; pH was adjusted to 7.25 using CsOH.

In current-clamp mode, action potentials were evoked by a 4 ms, 150 pA injection of depolarizing current pulses. The time from the initiation of action potential until the membrane potential returned to within 10 mV of the resting potential was deemed to action potential duration.

### Ca^2+^ Imaging Technology

Islet β cells were cultured on coverslips in RPMI 1640 medium for 3 h before the experiments. Cells were exposed to 2 μM Fura2-AM (Dojindo Laboratories, Japan) in KRBH buffer containing 2.8 mM glucose at 37°C for 30 min, then washed twice with buffer containing 2.8 mM glucose. The coverslips with cells were placed into a chamber mounted on the specimen stage of an inverted fluorescence microscope (Olympus Life Science, Japan). Intracellular Ca^2+^ was measured with dual wavelength excitation microspectrofluorimetry. With the excitation fluorescence wavelengths at 340 nm/380 nm and the emission fluorescence wavelength at 510 nm, the intracellular Ca^2+^ concentration is reflected by 340/380 ratio values (F340/F380) with MetaFluor software 7.8 (Molecular Devices). The concentration of S6 used in this protocol is 10 µM. All imaging experiments were performed in the dark at a temperature of 30°C.

### Statistical Analysis

Data were analyzed with SigmaPlot software (12.5 version) and presented as Mean ± SEM. Statistical analysis (Student’s t-test or one-way ANOVA tests) was performed and *p* < 0.05 was considered to be statistically significant.

## Results

### Virtual Screening and Calculation of Physical and Chemical Properties

**FIGURE 1:**
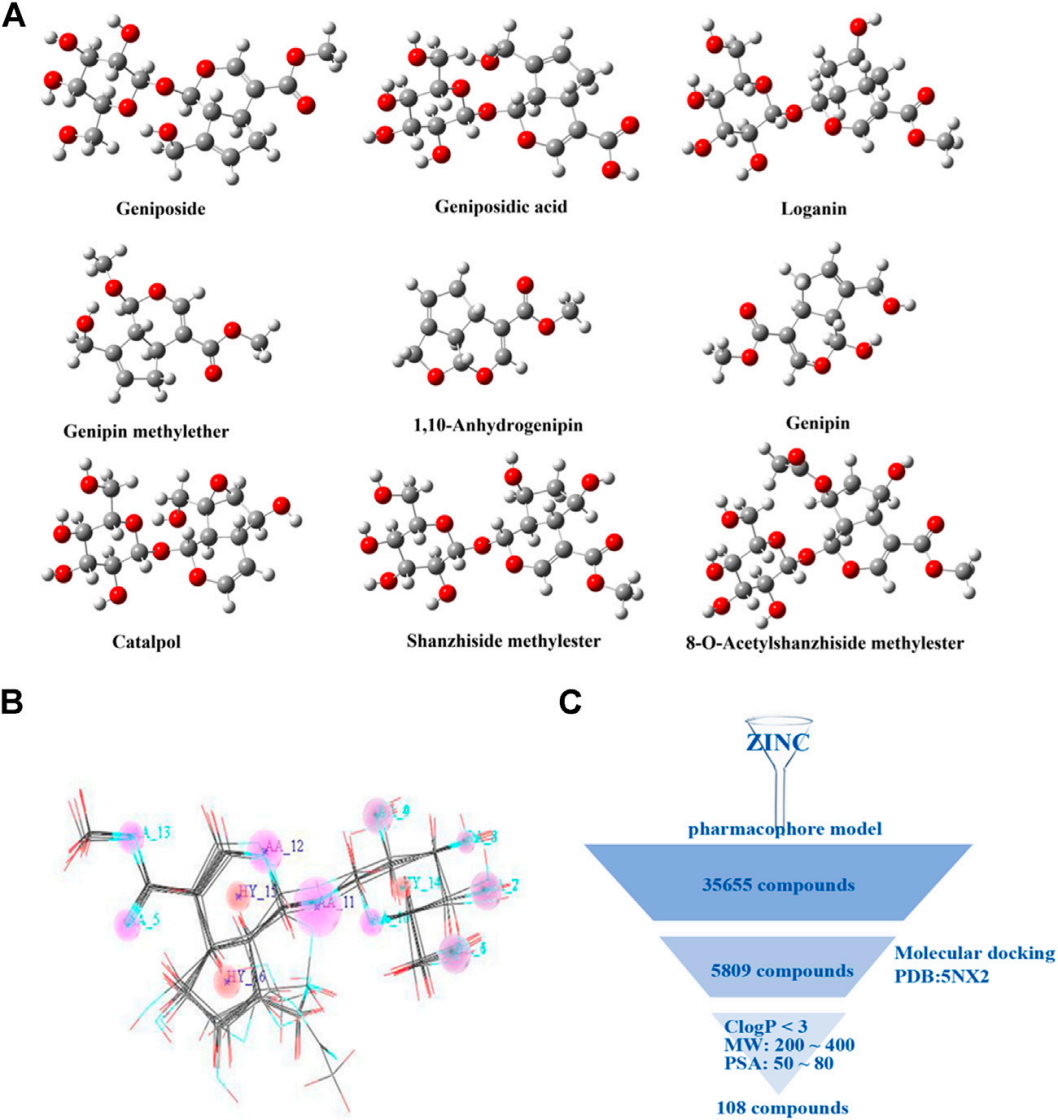
Virtual screening of small molecule compounds. **(A)** Chemical structures of reported active molecules. **(B)** Pharmacophore model obtained from nine compounds. **(C)** Virtual screening workflow.

The dataset used to generate the pharmacophore model comprises of nine reported GLP-1R agonists (Gong et al., 2014; Zhu et al., 2014). The geometric structures of reported compounds were optimized at the B3LYP/6-311G (d, p) level *via* Gaussian 16. Figure 1A presents the equilibrium structures of the nine compounds. The pharmacophore model generation with GALAHAD (Richmond et al., 2006; Shepphird and Clark, 2006) was integrated in SYBYL (Figure 1B). The pharmacophore models were used as a query to screen the ZINC 7.0 drug-like database (Irwin and Shoichet, 2005) (1.3 million compounds). We subjected 35,655 compounds obtained from this step to second step virtual screening based on molecular docking using Surflex-Dock in SYBYL. The GLP-1R X-ray crystal structure (PDB code 5NX2) was download from the Protein Data Bank, water was removed, and hydrogen atoms were added. The hits included 5809 compounds. For a virtual screening workflow see Figure 1C.

The selected compounds were further screened. The physicochemical properties of these compounds were calculated. Based on the physicochemical properties of oral drugs, the selection criteria here are set to: lipophilicity (C Log P) <3, molecule weight (MW) in 200∼400, and molecular polar surface area (PSA) in 50∼80 Å^2^ (Congreve et al., 2003; Gleeson, 2008; Lepre, 2011). The final 108 compounds met the requirements.

### Screening and Identification of Small Molecule GLP-1R Agonist S6

**FIGURE 2:**
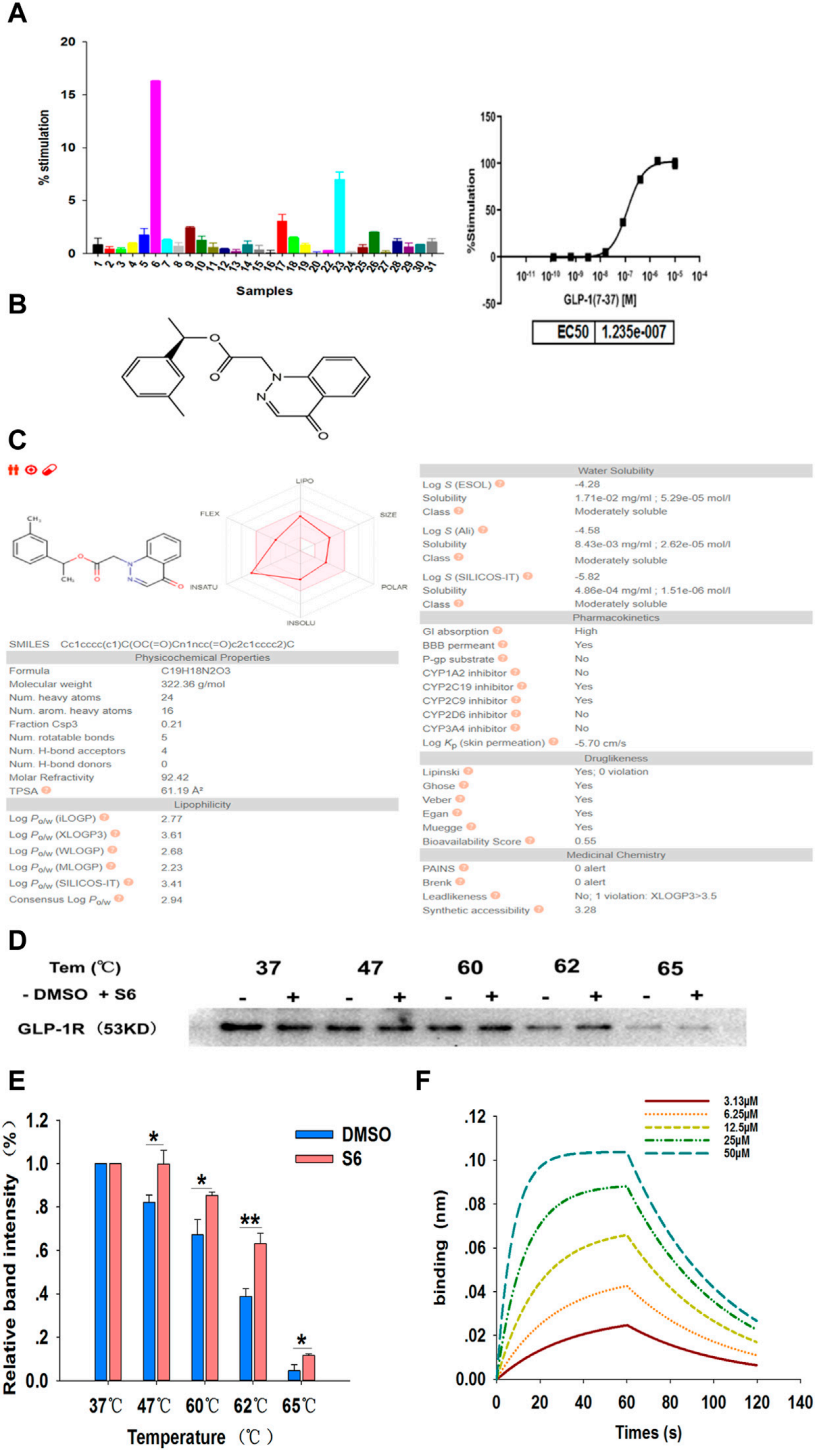
Screening and identification of small molecule GLP-1R agonist S6 through FLIPR-based calcium assays and CETSA. **(A)** The intracellular Ca^2+^ signals in CHO-K1/GLP-1R/Gα15 cells were measured using Calcium 4 Assay Kit, with GLP-1 (7-37) as a positive control (The maximum concentration was 10 μM, 5-fold dilution, 8-point concentration). The average fluorescence intensity value in the first 20 s is considered as the baseline level, the maximum fluorescence intensity value from 21 to 120 s minus the minimum fluorescence intensity value from 21 to 120 s is expressed as the relative fluorescence intensity value (ΔRFU). The activation percentage was calculated according to the following equation: % stimulation rate = (ΔRFU compounds—ΔRFU background)/(ΔRFU positive control—ΔRFU background) × 100%. Data are expressed as the mean ± SD, n = 3. **(B)** The chemical structure of S6 (1-(3-methylphenyl)ethyl 2-(4-oxo-1,4-dihydrocinnolin-1-yl)acetate), Molecular formula C_19_H_18_N_2_O_3_; Molecular weight is 322.364. **(C)** ADME profiling for the compound S6 using SwissADME. **(D)** CETSA for cell lysate treated with DMSO and S6 (10 μM) were conducted using western blotting to detect the interaction between S6 and GLP-1R, and quantification of this data is shown in **(E)**. Data are expressed as the mean ± SEM and compared by one-way ANOVA. n = 3, *p < 0.05, **p < 0.01. **(F)** Binding profiles of S6 to GLP-1R measured by BLI in OctetRED96. Binding kinetics was fit to 1:1 binding model by SigmaPlot 12.5.

The *in vitro* activity of the 30 commercially available compounds from virtual screening was first detected using the FLIPR-based calcium flux assay. In this screening system, changes in (Ca^2+^)_i_ were detected in CHO-K1/GLP-1R/Gα15 cells, thereby assessing the GLP-1R agonist property of test compounds. From *in vitro* screening, when the concentration of test samples is 10 μM, 16 out of 30 novel samples induced calcium responses (Figure 2A). As S6 (ZINC 71914349, Figure 2B) possessed the highest potency, it was chosen as the potential GLP-1R agonist for further experimental investigation.

To further assess whether the S6 has the potential to be oral, SwissADME (Daina et al., 2017) was used to compute physicochemical descriptors as well as to predict ADME parameters, pharmacokinetic properties, druglike nature and medicinal chemistry friendliness of S6. As we can see in Figure 2C, gastrointestinal absorption of S6 is high, which confirms again that S6 is a potential small molecule compound for oral use.

Cellular thermal shift assay (CETSA) is a recently developed approach to directly evaluate drug-target relationship (Molina et al., 2013). If a compound binds with the target protein, the thermal stability will increase (Jafari et al., 2014). In this study, we assessed the binding of S6 to GLP-1R in cell lysates. As showed in the gradient heating results, the stability of S6 to GLP-1R was superior to the DMSO-treated group, especially at 62°C (Figures 2D,E). Thus, the results demonstrated that S6 could specifically bind to GLP-1R.

To further confirm this result, we measured the binding affinities of S6 and GLP-1R using Bio-Layer Interferometry (BLI). The basic principle of BLI is by loading on a few protein sample on the surfaces of biosensor, and analyze the optical changing signals reflected from the biosensor surfaces (Wallner et al., 2013). As shown in Figure 2F, our results clearly demonstrated that S6 bound potently to GLP-1R (the dissociation constant (K_D_) value as 1.0 × 10^−5^ M) and showed a concentration-dependent manner.

### S6 Augments Insulin Secretion and [Ca^2+^]_i_ Levels by Activating the GLP-1R

**FIGURE 3:**
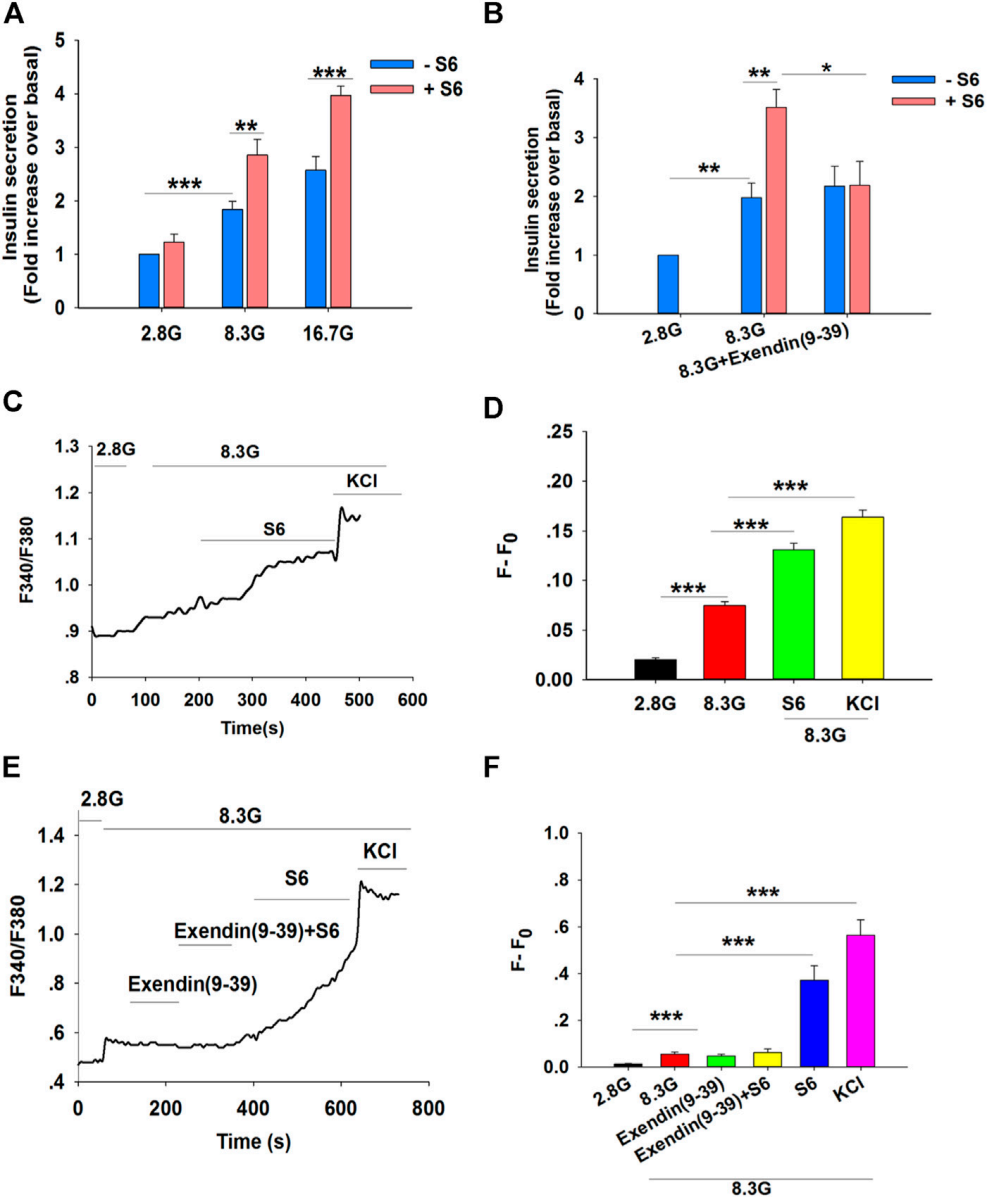
S6 stimulates insulin secretion and [Ca^2+^]_i_ levels by activating the GLP-1R. **(A)** The effect of S6 on insulin secretion from rat islets under different glucose conditions. Islets were treated with 10 μM S6 under 2.8 mM glucose (2.8 G), 8.3 mM glucose (8.3 G) and 16.7 mM glucose (16.7 G) conditions. Data are expressed as the mean ± SEM and compared by one-way ANOVA. n = 7, **p < 0.01, ***p < 0.001. **(B)** Effect of Exendin (9-39) on S6-induced insulin secretion. Islets were exposed to 10 μM S6 in the presence or absence of Exendin (9-39) (100 nM). Data are expressed as the mean ± SEM and compared by one-way ANOVA. n = 6, *p < 0.05, **p < 0.01. All data are normalized to basal secretion at 2.8 G. **(C)** Effect of S6 on [Ca^2+^]_i_ in the presence of 8.3 G in β cells. The changes in [Ca^2+^]_i_ were plotted by the ratio of 340/380 nm fluorescence. **(D)** The mean value of F-F_0_ in response to S6 as indicated (F: the fluorescence mean value within 30 s (15 s before and after the peak of F340/F380) after different treatments; F_0_: the fluorescence mean value within 30 s (15 s before and after the nadir of F340/F380) for 2.8 G). KCl (60 mM) was used as positive control. Data are expressed as the mean ± SEM and compared by one-way ANOVA. n = 13, ***p < 0.001. **(E)** Effect of Exendin (9-39) on S6-induced [Ca^2+^]_i_ in the presence of 8.3 G. The changes in [Ca^2+^]_i_ were plotted by the ratio of 340/380 nm fluorescence under different interventions. **(F)** The average value of F-F_0_ in response to S6 in the presence or absence of Exendin (9-39) as indicated. KCl (60 mM) was used as positive control. Data are expressed as the mean ± SEM and compared by one-way ANOVA. n = 8, ***p < 0.001.

A bona fide small molecule GLP-1R agonist would stimulate insulin secretion only in conditions of elevated glucose concentrations, and so the insulinotropic effect of S6 *in vitro* was assessed in pancreatic islets isolated from Wistar rats. In this assay, islets were exposed to S6 in different glucose concentrations (2.8, 8.3, or 16.7 mM). S6 stimulated insulin secretion in high (8.3 or 16.7 mM) glucose concentrations, but not at 2.8 mM glucose (Figure 3A), which suggested that S6 induced insulin release in a glucose-dependent manner.

To further evaluate S6, assays were performed using Exendin (9–39) (100 nM), an antagonist of GLP-1R. As predicted, S6-enhanced insulin secretion was blocked by Exendin (9–39) (Figure 3B). Taken together, our data support the conclusion that S6 is a GLP-1R agonist.

An increase in [Ca^2+^]_i_ is essential to trigger insulin exocytosis (Ashcroft et al., 1994). Therefore, the fluorescent calcium indicator Fura2-AM was used to monitor the changes in [Ca^2+^]_i_ in primary islet β cells. The ratio of 340/380 nm fluorescence was markedly increased in the presence of 8.3 mM glucose compared with control in basal glucose (2.8 mM). A further enhancement of the 340/380 nm fluorescence ratio was observed following treatment with S6 (10 μM) compared with 8.3 mM glucose conditions (Figures 3C,D).

To further verify whether S6 increased [Ca^2+^]_i_ *via* GLP-1R activation, β cells were treated with Exendin (9–39). Similarly, S6-induced [Ca^2+^]_i_ elevation was attenuated by Exendin (9–39) (Figures 3E,F). These findings suggested that S6 evoked a dramatic increase in [Ca^2+^]_i_ upon GLP-1R activation, resulting in enhanced insulin secretion.

### S6 Does Not Directly Alter Voltage-dependent Ca^2+^ Channels in β Cells, but Inhibits Kv Channels and Prolongs the Action Potential Duration

**FIGURE 4:**
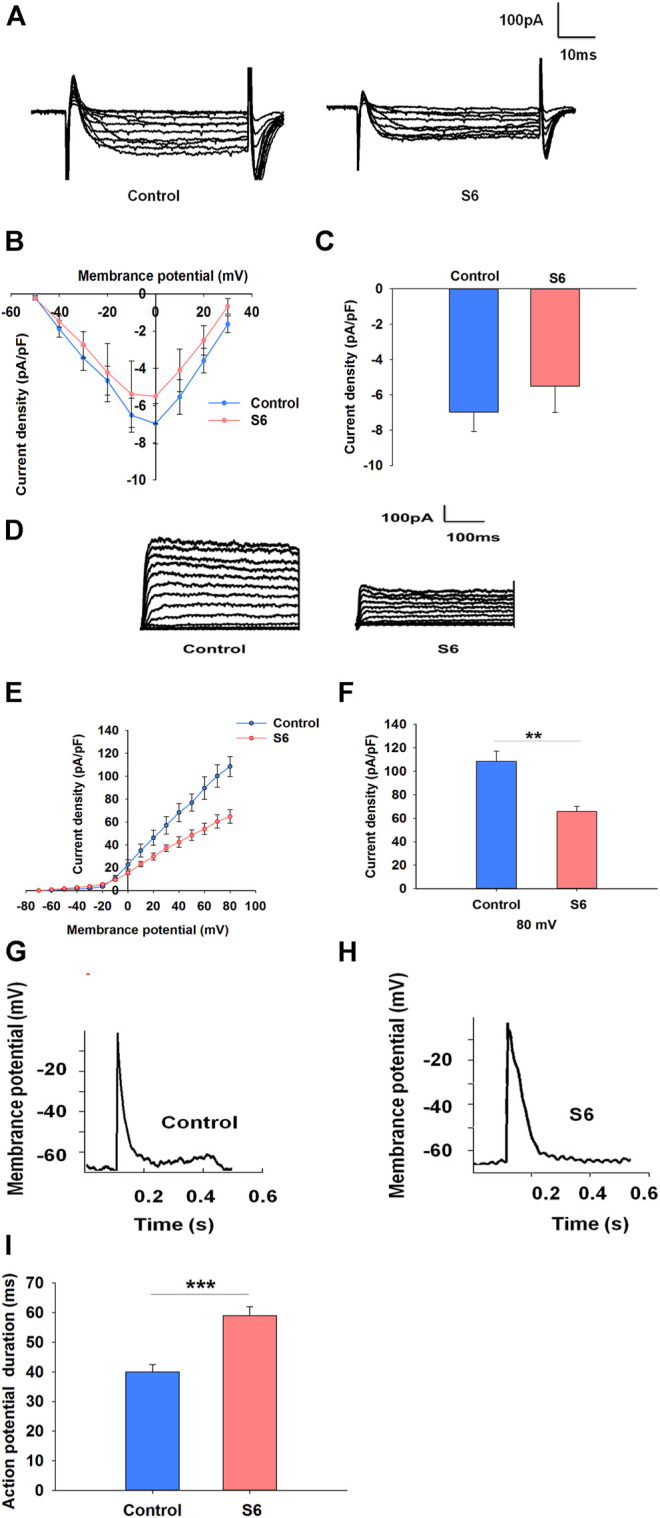
Effects of S6 on voltage-dependent Ca^2+^ channels, Kv channels and action potential duration in rat β-cells. (1) Effect of S6 on voltage-dependent Ca^2+^ channels. Ca^2+^ currents were recorded in the conventional whole-cell configuration from a holding potential of −70 mV to various depolarizing pulses (−50 to +30 mV) in 10 mV steps. **(A)** Typical inward Ca^2+^ current traces recorded with and without S6. **(B)** Current–voltage relationship curves for Ca^2+^ currents recorded with and without S6. **(C)** Summary of the average current density recorded at 0 mV depolarization. Data are expressed as the mean ± SEM and compared by one-way ANOVA. n = 7. (2) Effects of S6 on Kv channels and action potential duration. Kv currents were recorded from a holding potential of −70 mV to various depolarizing voltages (−70 to +80 mV). **(D)** Representative outward K^+^ current traces recorded in the presence or absence of S6. **(E)** Current–voltage relationship curves of Kv channels. **(F)** Summary of the average current density of Kv channels recorded at 80 mV depolarization. Data are expressed as the mean ± SEM and compared by one-way ANOVA. n = 7, **p < 0.01. (3) Action potentials were elicited by applying 4 ms, 150 pA current injections. Representative action potential waveforms are shown for β cells stimulated without **(G)** or with S6 **(H)**. **(I)** Summary of the average action potential durations. Data are expressed as the mean ± SEM and compared by one-way ANOVA. n = 7, ***p < 0.001.

Voltage-dependent Ca^2+^ channels are the main entrance for extracellular Ca^2+^ influx in β cells (Jacobson and Shyng, 2020). To characterize whether S6 directly activated voltage-dependent calcium channels, we performed whole-cell patch clamp experiments. Representative Ca^2+^ current traces were obtained by a series of 10 mV depolarizing steps (−50– +30 mV) from a holding potential of −70 mV. (Figure 4A). The current–voltage relationship curves reflected the effects of S6 on voltage-dependent calcium channels compared with control (Figure 4B). The data indicated that Ca^2+^ currents were not significantly affected after application of S6 (−6.9781 ± 1.0858) compared to that in the control (−5.5022 ± 1.4896) at the current density of 0 mV (Figure 4C).

Kv channels are activated by pancreatic β cells depolarization. Inhibition of Kv channels can extend action potential duration, resulting in the elevation of intracellular Ca^2+^ concentration and insulin secretion (Macdonald and Wheeler, 2003; Rorsman and Ashcroft, 2018). Accordingly, we aimed to observe whether S6 affected Kv channels as well as action potential duration. As expected, S6 significantly decreased the current densities to 65.65 ± 4.68 pA/pF from 108.57 ± 8.71 pA/pF (Figures 4D–F). Likewise, action potential duration was significantly prolonged after treatment with S6 compared to control (Figures 4G–I).

### S6 Inhibits Kv Channels *via* Modulating GLP-1R

**FIGURE 5:**
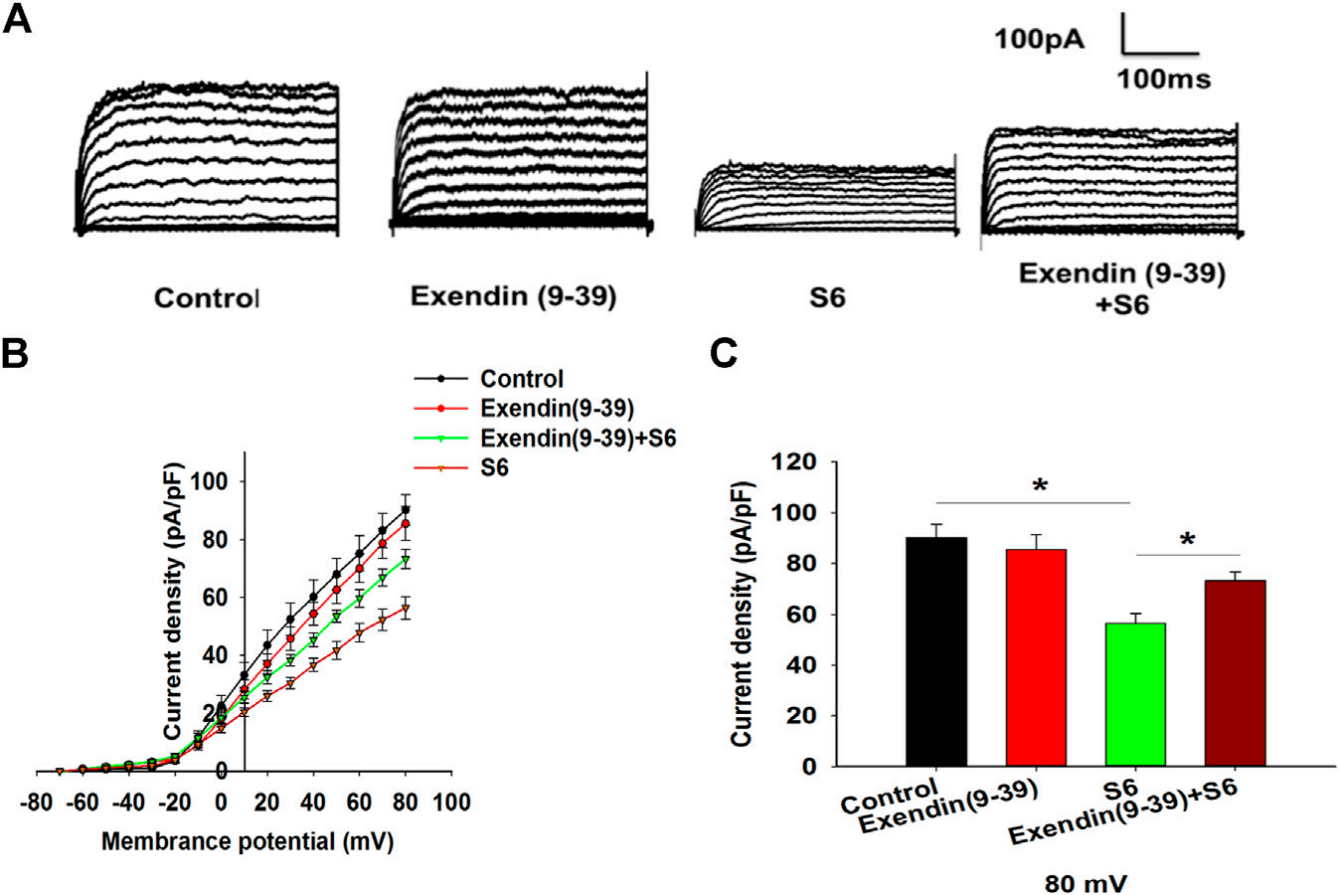
S6 inhibits Kv channels via modulating GLP-1R. **(A)** Representative current traces recorded under the different interventions. **(B)** Current-voltage relationship curves of Kv channels. **(C)** Summary of the mean current density of Kv channels recorded at 80 mV depolarization. Data are expressed as the mean ± SEM and compared by one-way ANOVA. n = 7, *p < 0.05.

To further observe the relationship between S6-inhibited Kv channels and GLP-1R activation, experiments were performed using Exendin (9–39). The data showed that Kv currents were not affected by Exendin (9–39) alone; however, Exendin (9–39) reversed the inhibitory effect of S6 on Kv currents (Figures 5A–C). Therefore, these data indicate that S6 inhibits Kv currents *via* GLP-1R.

## Discussion

Type 2 diabetes mellitus is mainly caused by two physiological and pathological defects, i.e. insufficient insulin secretion together with insulin resistance (CERTARA, 2014). GLP-1R is a potent target for the treatment of type 2 diabetes mellitus (Redij et al., 2019), and small molecule agonists targeting GLP-1R can provide several potential benefits and overcome defects associated with peptide drugs. Previously, small molecule drug discovery efforts were performed by high-throughput screening. In the present study, we attempt to use another efficient approach, virtual screening, to develop novel and potent small molecule GLP-1R agonists.

The rational strategy used for virtual screening was to design a pharmacophore model (Yang, 2010) from nine structurally known small molecule GLP-1R agonists. Employing the generated 3D model and performing screening in the ZINC database, we attained a series of compounds as potential agonists of GLP-1R. In addition, good oral bioavailability is usually a vital consideration in the field of oral GLP-1R agonist development (Willard et al., 2012). We hence evaluated the possibility of small molecule compounds as potential oral drugs and identified 108 potential GLP-1R agonists for further experimental study.

GLP-1R belongs to seven transmembrane spanning G-protein-coupled receptors (GPCRs) (Baggio and Drucker, 2007). Nowadays, cell-based calcium flux assays on FLIPR fluorometric imaging detection systems have become increasingly useful to study GPCRs (Gopalakrishnan et al., 2005; Miret et al., 2005; Siehler, 2008). The agonistic effects of compounds on GPCRs can be assayed by their ability to raise intracellular Ca^2+^. This is particularly suitable for initial identification of novel lead compounds (Vetter, 2012). The activation of GPCRs signaling *via* the Gαq subunits, such as Gα15 and Gα16 (Offermanns and Simon, 1995), activates phospholipase C-β, leading to the hydrolysis of phosphatidylinositol 4,5-bisphosphate, yielding diacylglycerol and inositol 1,4,5-trisphosphate, where the latter triggers the release of calcium (Offermanns and Simon, 1995). In this experiment, we monitored the response of (Ca^2+^)_i_ in CHO-K1/Gα15/GLP-1R cells for further screening of the GLP-1R-agonistic activity of small molecule compounds. Our data clearly showed that S6 exerted the strongest potential in promoting the increase of (Ca^2+^)_i,_, which suggested that S6 likely functions as a potent small molecule GLP-1R agonist, although further experimental verification is needed.

In the process of drug discovery, confirmation of in-cell target engagement is an essential step (Jensen et al., 2015). We first introduced CETSA to monitor and quantify the extent to which a drug reaches and directly binds to a protein target. This is regarded as the preferred method for determining drug-target engagement in the cell (Jensen et al., 2015). When treating cells with a drug, unbound proteins will denature and precipitate at elevated temperatures, whereas ligand-bound ones will stabilize and remain in solution. In the present study, we confirmed that S6 binds to GLP-1R with affinities shown by the results of CETSA, which is consistent with the previous conclusion. In addition, we further investigated the interaction between S6 and GLP-1R by BLI. It is an emerging optical technique and also widely used in drug screening for detecting the binding of biomolecular interactions. Finally, we indeed ascertained the strong binding affinity between S6 and GLP-1R. Taken together, these two results further confirm our original conjecture that S6 is a potential and effective GLP-1R agonist.

The key to confirm S6 as a bona fide small molecule GLP-1R agonist is that S6 induced insulin secretion from pancreatic β cells in a glucose-dependent fashion. This is further reinforced by the observation that the GLP-1R antagonist Exendin (9–39) blocked S6-induced insulin secretion. Consequently, the vital glucose-dependent insulinotropic effects of GLP-1R activation are maintained by S6.

Research has suggested that changes in (Ca^2+^)_i_ play a prominent role in the regulation of insulin release from islet β cells (Meloni et al., 2013). As expected, our calcium imaging results showed that S6 induced a dramatic increase in (Ca^2+^)_i_ *via* GLP-1R in rat pancreatic β cells. This is consistent with the results of our secretion assay. In excitatory cells, elevated cytosolic Ca^2+^ is regulated by a series of electrical activity (Rorsman and Ashcroft, 2018). The increase in (Ca^2+^)_i_ responsible for glucose-dependent insulin secretion is mainly controlled by voltage-dependent Ca^2+^ channels (Ashcroft et al., 1994; Rorsman and Ashcroft, 2018). Beyond that, blocking Kv channels is another pathway to potently promote insulin secretion (Jacobson and Shyng, 2020). Although our data showed that S6 failed to have a direct effect on voltage-dependent Ca^2+^ channels, S6 inhibited Kv channels in a GLP-1R-dependent manner and prolonged the action potential duration, allowing a longer period of Ca^2+^ influx through voltage-dependent Ca^2+^ channels and ultimately enhancing insulin secretion.

In conclusion, this research demonstrates that S6, as a potential and oral GLP-1R agonist, enhances glucose-dependent insulin secretion *in vitro via* GLP-1R/Kv/Ca^2+^. Our findings validate the significance of the rational design approach in GLP-1R drug discovery. Although further explorations are necessary, the discovery of S6 still serves as an implication for the future development of small molecule GLP-1R agonists.

## Data Availability

The original contributions presented in the study are included in the article/Supplementary Material, further inquiries can be directed to the corresponding authors.
